# Prevalence and Outcomes of Gastrointestinal Manifestations in an Australian Scleroderma Cohort

**DOI:** 10.1002/acr.25426

**Published:** 2024-10-04

**Authors:** Alannah Quinlivan, Dylan Hansen, Wendy Stevens, Laura Ross, Nava Ferdowsi, Susanna M. Proudman, Jennifer G. Walker, Joanne Sahhar, Gene‐Siew Ngian, Diane Apostolopoulos, Lauren V. Host, Gabor Major, Chamara Basnayake, Kathleen Morrisroe, Mandana Nikpour

**Affiliations:** ^1^ St. Vincent's Hospital Melbourne, Fitzroy, and The University of Melbourne Melbourne Victoria Australia; ^2^ St. Vincent's Hospital Melbourne Fitzroy Victoria Australia; ^3^ Royal Adelaide Hospital and the University of Adelaide Adelaide South Australia Australia; ^4^ Royal Adelaide Hospital and Flinders University, Adelaide, Flinders Medical Centre Bedford Park South Australia Australia; ^5^ Monash Health and Monash University Clayton Victoria Australia; ^6^ Fiona Stanley Hospital Murdoch Western Australia Australia; ^7^ John Hunter Hospital, New Lambton Heights, University of Newcastle Callaghan New South Wales Australia; ^8^ St. Vincent's Hospital Melbourne, Fitzroy, The University of Melbourne, Melbourne, Victoria, and the University of Sydney and Royal Prince Alfred Hospital Camperdown New South Wales Australia

## Abstract

**Objective:**

The gastrointestinal tract (GIT) is the most commonly affected internal organ in systemic sclerosis (SSc). We sought to determine the prevalence and impact of GIT symptoms on survival and patient‐reported outcomes.

**Methods:**

A total of 907 consecutive patients from the Australian Scleroderma Cohort Study who had prospectively completed the University of California, Los Angeles, Scleroderma Clinical Trials Consortium Gastrointestinal Tract 2.0 Questionnaire (UCLA GIT) between 2015 and 2021 were included. The associations between UCLA GIT scores and physical function (Scleroderma Health Assessment Questionnaire), quality of life (QoL; Short Form 36), mood (Patient‐Reported Outcomes Measurement Information System [PROMIS] anxiety and depression domains), fatigue (Functional Assessment of Chronic Illness Therapy–Fatigue score), and employment were investigated using multivariable population‐averaged panel models using generalized estimating equations (GEEs). Kaplan‐Meier curves and multivariable Cox proportional hazard regression models were used to evaluate survival according to total UCLA GIT scores.

**Results:**

GIT symptoms were reported in 87% of participants, with 46% to 52% reporting moderate to very severe symptoms of reflux, distension, diarrhea, and constipation. Higher total UCLA GIT scores were associated with worse QoL, physical function, fatigue, anxiety, and depression (*P* < 0.001). In the multivariable GEE analysis, moderate and severe to very severe total scores, reflux scores, and distension scores were associated with worse physical function, QoL, fatigue, anxiety, and depression compared to mild scores (*P* < 0.05). Patients with severe total scores and diarrhea scores were more likely to be unemployed compared to those with mild scores (*P* < 0.05). UCLA GIT total scores were not independently associated with death in our cohort.

**Conclusion:**

GIT manifestations are common in SSc and negatively impact QoL, physical function, and employment but are not directly associated with increased death.

## INTRODUCTION

Systemic sclerosis (SSc) or scleroderma is a multiorgan autoimmune disease of the connective tissues characterized by pathogenic processes of inflammation, vasculopathy, and fibrosis.^1^ Although rare, SSc can have devastating consequences to the patient regarding death and quality of life (QoL).^2^ Although disease manifestations vary greatly among patients with SSc, the gastrointestinal tract (GIT) remains the most commonly affected internal organ, with up to 90% of patients with SSc reporting GIT symptoms.^3^ Any part of the GIT, from mouth to anus, can be affected.^4^ GIT involvement in SSc has been shown in various studies to have a significant impact on QoL, physical function, mood, fatigue, and sleep.^5^, ^6^, ^7^, ^8^, ^9^, ^10^, ^11^, ^12^ In its most severe form, GIT complications (such as malabsorption and pseudo‐obstruction) can also negatively impact survival.^13^, ^14^

**SIGNIFICANCE & INNOVATIONS**

- Gastrointestinal manifestations affect 87% of Australian patients with scleroderma.
- Reflux and distension were the symptoms most significantly associated with worse measures of quality of life, physical function, fatigue, and mood in patients with scleroderma.
- Patients with severe gastrointestinal symptoms and severe diarrhea were more likely to be unemployed compared to those with mild symptoms.

The University of California, Los Angeles, Scleroderma Clinical Trials Consortium Gastrointestinal Tract 2.0 Questionnaire (UCLA GIT) is a patient‐reported outcome (PRO) measure that has been validated in SSc and translated across many languages.^15^, ^16^ It is one of the most commonly used tools to measure GIT symptoms in SSc and provides a total score as well as scores in five symptom domains (reflux, distension, diarrhea, constipation, and fecal incontinence [FI]) and two QoL domains (emotional and social well‐being). The aim of this study was to determine the prevalence and impact of GIT symptoms on QoL, physical function, employment, and survival in Australian patients with SSc.

## PATIENTS AND METHODS

### Patients

Consecutive patients with SSc from the Australian Scleroderma Cohort Study (ASCS) who had completed the UCLA GIT at least once were included in this analysis. The ASCS, a prospective multicenter cohort study of risk and prognostic factors of SSc, was established in 2007. Patients with SSc who fulfill the 2013 American College of Rheumatology and EULAR criteria^17^ are recruited and undergo yearly screening for pulmonary arterial hypertension (PAH) and interstitial lung disease (ILD), with demographic and disease data collected at this time. Annual questionnaires of PRO measures, including physical function, QoL, and fatigue, are collected yearly (see Data collection section). Each participant gives consent to be part of the ASCS, with ethical approval obtained from all the participating study sites.

### Data collection

Data collection occurred between 2015 and 2021. Deidentified data collected annually as part of the ASCS include demographics, disease features and complications, death, and PRO measurements of QoL, mood, fatigue, and physical function. Demographic data includes age, sex, and employment. SSc‐related data includes disease duration (defined as the time since onset of first non–Raynaud phenomenon manifestation), disease subtype, medications, autoantibody status, and disease complications.

Complications of SSc include ILD, PAH, myocardial disease, myositis, Raynaud phenomenon, and digital ulcers. ILD is defined by the presence of findings characteristic of pulmonary fibrosis on high‐resolution computed tomography (HRCT) of the chest, with severity defined as fibrosis affecting <20% of the lung fields (mild), 20% to 30% of the lung fields (moderate), and >30% of the lung fields (severe). HRCT scans are performed in patients in whom there is clinical suspicion of ILD (based on clinical examination or pulmonary function testing). PAH is diagnosed by right‐sided heart catheterization (RHC) using international criteria.^18^ Patients were referred for RHC based on elevated right ventricular systolic pressure or signs suggestive of PAH on the screening transthoracic echocardiogram. Myositis is defined as the presence of clinically evident weakness, laboratory findings of elevated creatine kinase levels, and evidence of muscle inflammation (on magnetic resonance imaging, electromyography, or muscle biopsy). Myocardial disease is defined as the presence of systolic or diastolic dysfunction or rhythm disturbances attributable to SSc determined by a specialist with experience in SSc. “Mortality” included scleroderma‐associated death and all‐cause death.

PRO measures are collected annually using validated questionnaires. For this study, physical function was measured using the Scleroderma Health Assessment Questionnaire (SHAQ), whereas QoL was measured using the Short Form 36 (SF‐36). Mood was assessed using Patient‐Reported Outcomes Measurement Information System (PROMIS) anxiety and depression domains. Functional Assessment of Chronic Illness Therapy–Fatigue (FACIT‐Fatigue) scores were used as a measure of fatigue. Employment data were collected yearly from patients who were considered to be employed if they reported full‐time or part‐time employment or full‐time study. Patients were unemployed if they reported unemployment or disability preventing work. Patients who were retired or reported home duties were removed from the multivariable analysis.

GIT symptoms were measured by the UCLA GIT, which has been collected yearly from all patients in the ASCS. The questionnaire is divided into five symptom domains (reflux, distension, diarrhea, constipation, and FI) and two QoL domains relating to the effect of GIT symptoms on patients’ social function and emotional well‐being. Scores are provided for each domain, as well as a total score, based on previous validation studies,^19^ with higher scores denoting worse disease. Scores were grouped into the following three categories: “none to mild,” “moderate,” and “severe to very severe.” The total score is calculated as a composite of all domains,^16^ excluding constipation, with scores ranging from 0 to 3.

### Statistical analysis

All statistical analyses were performed using STATA 15.1 (StataCorp LP). Data are presented as median (interquartile range [IQR]) for non‐normally distributed continuous variables, and as number (percentage) for categorical variables. Differences in frequency were tested using chi‐square and Fisher's exact tests. For non‐normally distributed continuous variables, the *P* value was calculated using the Kruskal‐Wallis equality‐of‐populations rank test (for three categories) and Wilcoxon rank sum test (for two categories). A *P* value of <0.05 was considered statistically significant. Univariate analysis was performed using the worst ever recorded UCLA GIT scores. Multivariable population‐averaged panel models using generalized estimating equations (GEEs) were used to determine the associations between UCLA GIT scores and QoL (using SF‐36 physical and mental component scores), mood (using PROMIS anxiety and depression domains), physical function and disability (using SHAQ), and fatigue (using FACIT‐Fatigue score) collected at each and every visit. Covariates in the multivariable model included sex, disease duration, SSc subtype, PAH, ILD, digital ulcers, Raynaud phenomenon, myocardial disease, and myositis.

Multivariable population‐averaged panel models using GEEs were also used to determine the association between UCLA GIT scores and unemployment using data collected at each visit. Covariates included sex, age, SSc subtype, PAH, ILD, digital ulcers, Raynaud phenomenon myocardial disease, and myositis. Hazard ratios were calculated using total UCLA GIT scores and symptom domain scores, including reflux, distension, diarrhea, and constipation. Because of the low number of patients with reported FI, this symptom domain was excluded from our multivariable models. The relationship between GIT symptoms and survival was analyzed using Kaplan‐Meier (K‐M) survival curves and multivariable Cox proportional hazard regression models (using “ever present” variables) with the proportional hazards assumption tested using Schoenfeld residuals. The datasets used and/or analyzed during the current study are available from the corresponding author on reasonable request.

## RESULTS

### Demographic data

**Figure 1:**
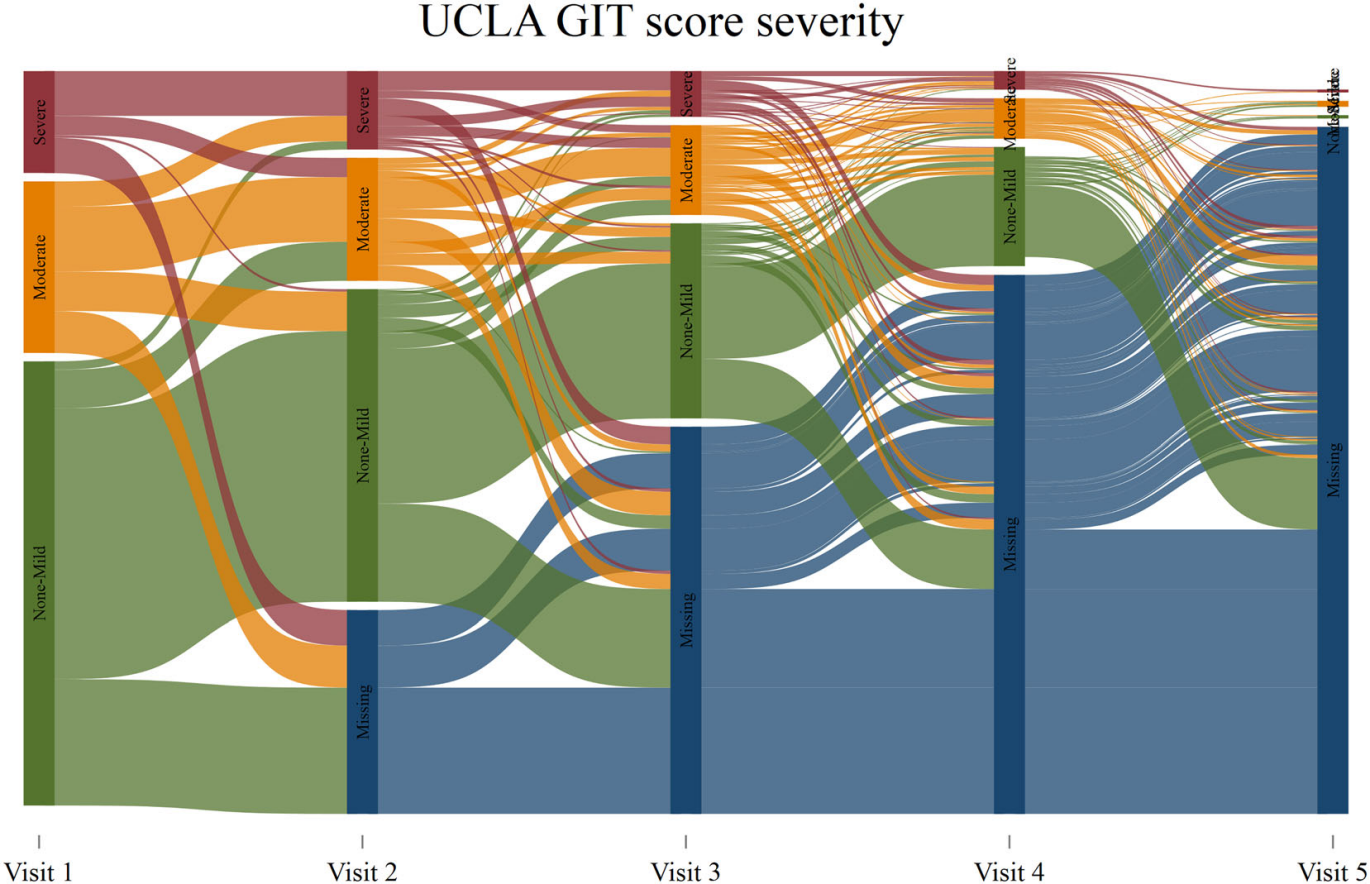
Sankey diagram of progression of UCLA GIT total severity score. FACIT, Functional Assessment of Chronic Illness Therapy; MCS, mental component score; PCS, physical component score; PROMIS, Patient‐Reported Outcomes Measurement Information System; SF36, Short Form 36; SHAQ, Scleroderma Health Assessment Questionnaire; UCLA GIT, University of California, Los Angeles, Scleroderma Clinical Trials Consortium Gastrointestinal Tract 2.0 Questionnaire.

Overall, 907 patients with SSc were included in this analysis (see Table 1). The average age of respondents was 46 (±14.4) years; 86.2% of participants were female, and 76.9% had the limited disease subtype. The mean disease duration was 14.2 years (SD 7.7–22.5 years). The average length of follow‐up was 2.0 years (SD 0–3.1 years), 144 (16.9%) were lost to follow‐up, and 53 (6.2%) died (Supplementary Table 1). There was no significant difference between current patients and those lost to follow‐up (Supplementary Table 2). Over the follow‐up period, the majority of participants’ scores (71%) did not change (Figure 1).

**Table 1:** Demographic and clinical characteristics by UCLA GIT total score*

Characteristics	All patients (N = 907)	None‐to‐mild score (n = 445)	Moderate (n = 231)	Severe to very severe score (n = 177)	*P*
Female, n (%)	733 (86.2)	365 (82.6)	203 (87.9)	165 (93.2)	0.002
Disease duration, mean (SD), y	14.2 (7.7–22.5)	12.75 (6.1–20.4)	14.2 (8.5–22.9)	16.8 (11.6–26.5)	<0.001
Disease subtype, n (%)					
Limited	611 (76.9)	316 (78.6)	171 (77.0)	124 (72.5)	–
Diffuse	184 (23.1)	86 (21.4)	51 (23.0)	47 (27.5)	0.29
Death (all‐cause mortality), n (%)	53 (5.8)	27 (6.1)	15 (6.5)	11 (6.3)	0.98
ANA centromere (+), n (%)	361 (44.73)	163 (39.5)	105 (47.7)	93 (53.4)	0.005
Scl‐70 (+), n (%)	134 (14.8)	78 (18.9)	33 (15.4)	23 (13.6)	0.24
U1 RNP (+), n (%)	56 (6.1)	36 (8.8)	10 (4.7)	10 (5.9)	0.14
Digital ulcers, n (%)^a^	415 (45.8)	187 (42.0)	111 (48.1)	117 (66.1)	<0.001
Calcinosis, n (%)^a^	147 (16.2)	65 (14.9)	37 (16.3)	45 (25.7)	0.006
Myositis, n (%)^a^	30 (3.3)	19 (4.3)	8 (3.5)	3 (1.7)	0.29
Myocardial disease, n (%)^a^	9 (0.9)	2 (0.4)	4 (1.7)	3 (1.7)	0.20
PAH, n (%)^b^	62 (7.3)	27 (6.1)	21 (9.1)	14 (7.9)	0.33
ILD, n (%)^c^	267 (34.5)	139 (34.5)	65 (30.7)	63 (39.4)	0.22
PPI use, n (%)^a^	702 (82.39)	324 (73.0)	207 (89.6)	171 (96.6)	<0.001
Employed, n (%)	342 (37.7)	207 (46.5)	91 (74.6)	44 (51.2)	<0.001
Pseudo‐obstruction, n (%)	29 (3.5)	5 (1.1)	6 (2.6)	18 (10.2)	<0.001
Malabsorption, n (%)	26 (3.3)	3 (0.7)	7 (3.3)	16 (9.5)	<0.001

* The categorical variables are compared with a *P* value, which was calculated using the chi‐square test for categorical variables and using the Kruskal‐Wallis equality‐of‐populations rank test for continuous variables. ANA, antinuclear antibody; ILD, interstitial lung disease; PAH, pulmonary arterial hypertension; PPI, proton pump inhibitor; SD, standard deviation; UCLA GIT, University of California, Los Angeles, Scleroderma Clinical Trials Gastrointestinal Tract 2.0 Questionnaire.

^a^ Ever recorded during follow‐up.

^b^ PAH was defined as mean PAP >20 mm Hg, a pulmonary capillary wedge pressure ≤15 mm Hg, and pulmonary vascular resistance >2 Woods units on right‐sided heart catheterization.

^c^ ILD was defined as the presence of characteristic pulmonary fibrosis on high‐resolution computed tomography of the chest.

GIT symptoms were common, with 46% to 52% of participants reporting moderate to very severe symptoms of reflux, distension, diarrhea, and constipation (Supplementary Table 3). Few patients reported moderate FI (9.4%), and none reported severe FI. No GIT symptoms were reported in 118 patients (13%). Female sex, longer disease duration, calcinosis, digital ulceration, pseudo‐obstruction, malabsorption, and anticentromere antibody were more common in patients with severe total GIT symptom scores on univariable analysis (*P* < 0.05; Table 1).

### QoL

**Figure 2:**
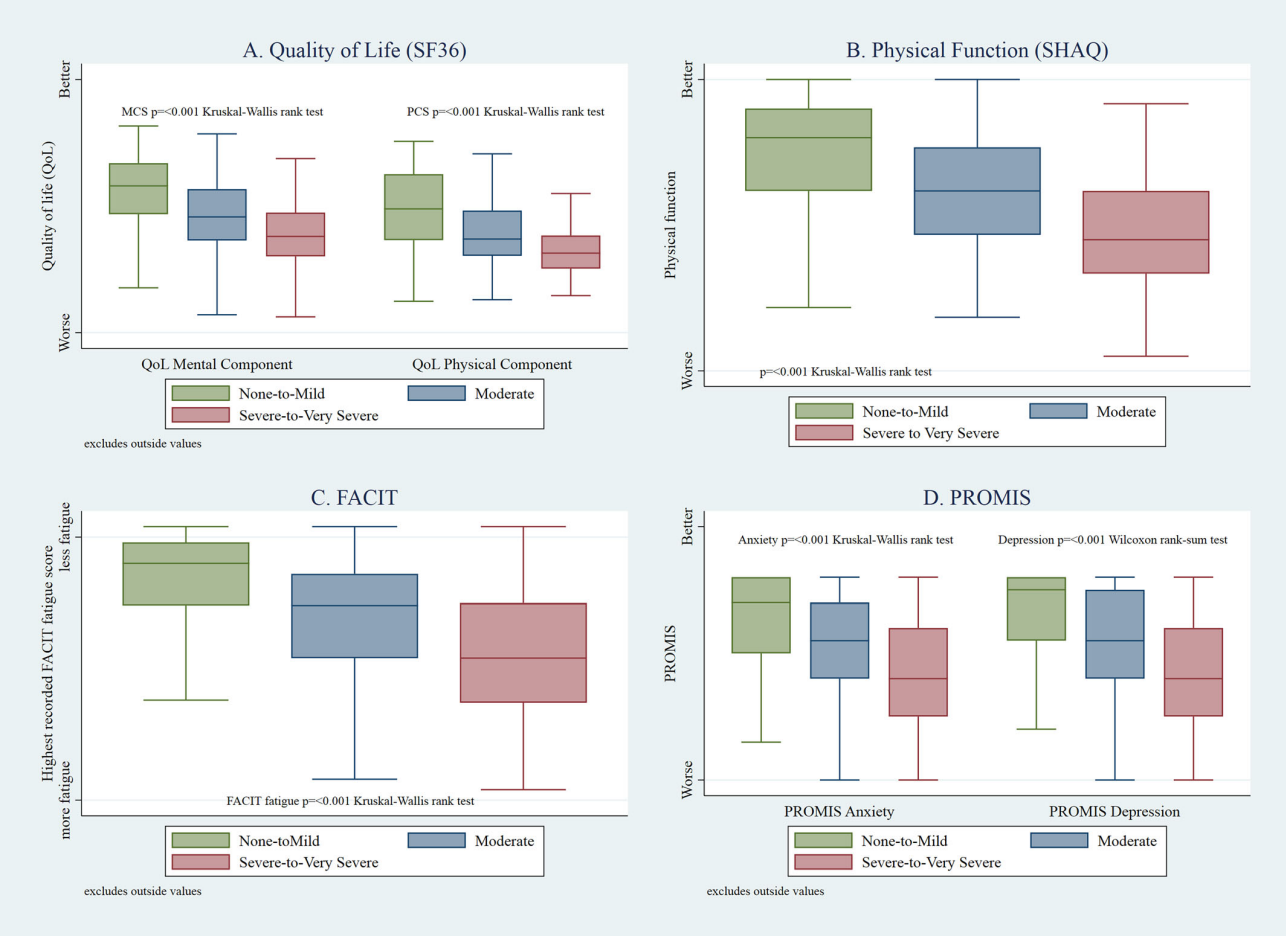
SF‐36, SHAQ, FACIT‐Fatigue, and PROMIS anxiety and depression by UCLA GIT total score severity. FACIT‐Fatigue, Functional Assessment of Chronic Illness Therapy–Fatigue; PROMIS, Patient‐Reported Outcomes Measurement Information System; SF‐36, Short Form 36; SHAQ, Scleroderma Health Assessment Questionnaire; UCLA GIT, University of California, Los Angeles, Scleroderma Clinical Trials Consortium Gastrointestinal Tract 2.0 Questionnaire.

Moderate and severe to very severe total UCLA GIT scores were significantly associated with worse QoL as measured by the SF‐36 physical and mental component scores (*P* < 0.001; see Figure 2) on univariate analysis. When looking at individual symptom domains, moderate and severe to very severe symptoms of reflux and distension were significantly associated with worse SF‐36 physical component scores on multivariable analysis (*P* < 0.003; see Table 2). Regarding SF‐36 mental component scores, severe to very severe scores in all symptom domains were associated with worse mental component scores on multivariable analysis (*P* < 0.001).

**Table 2:** Multivariable analysis of measures of QoL (SF‐36 PCS and MCS), physical function (SHAQ), fatigue (FACIT‐Fatigue), anxiety and depression (PROMIS) according to UCLA GIT symptom domain scores*

Variable and score	Coefficient	*P*	95% CI
SF‐36 PCS			
UCLA GIT reflux severity			
Moderate	−1.79	<0.001	−2.80 to −0.78
Severe to very severe	−4.76	<0.001	−6.26 to −3.26
UCLA GIT distention severity			
Moderate	−1.96	0.002	−3.22 to −0.69
Severe to very severe	−1.93	0.003	−3.21 to −0.66
UCLA GIT diarrhea severity			
Moderate	−0.59	0.18	−1.45 to 0.27
Severe to very severe	−1.19	0.13	−2.71 to 0.34
UCLA GIT constipation severity			
Moderate	−0.41	0.35	−1.28 to 0.46
Severe to very severe	0.02	0.97	−1.46 to 1.50
SF‐36 MCS			
UCLA GIT reflux severity			
Moderate	−2.21	<0.001	−3.41 to −1.02
Severe to very severe	−4.06	<0.001	−5.84 to −2.28
UCLA GIT distension severity			
Moderate	−1.07	0.12	−2.44 to 0.29
Severe to very severe	−4.85	<0.001	−6.40 to 3.30
UCLA GIT diarrhea severity			
Moderate	−1.19	0.05	−2.36 to −0.02
Severe to very severe	−2.68	0.008	−4.65 to −0.70
UCLA GIT constipation severity			
Moderate	−0.47	0.38	−1.51 to 0.57
Severe to very severe	−2.06	0.04	−4.07 to 0.06
SHAQ			
UCLA GIT reflux severity			
Moderate	3.30	<0.001	2.26 to 4.34
Severe to very severe	7.31	<0.001	5.72 to 8.89
UCLA GIT distention severity			
Moderate	1.92	0.002	0.71 to 3.13
Severe to very severe	3.93	<0.001	2.67 to 5.19
UCLA GIT diarrhea severity			
Moderate	1.76	<0.001	0.93 to 2.59
Severe to very severe	3.58	<0.001	2.11 to 5.06
UCLA GIT constipation severity			
Moderate	0.87	0.05	−0.01 to 1.75
Severe to very severe	2.27	0.006	0.66 to 3.88
FACIT‐Fatigue			
UCLA GIT reflux severity			
Moderate	−3.72	<0.001	−4.91 to −2.53
Severe to very severe	−6.33	<0.001	−8.21 to −4.45
UCLA GIT distention severity			
Moderate	−1.81	0.006	−3.11 to −0.51
Severe to very severe	−6.00	<0.001	−7.51 to −4.49
UCLA GIT diarrhea severity			
Moderate	−1.78	<0.001	−2.68 to −0.88
Severe to very severe	−2.07	0.02	−3.76 to −0.38
UCLA GIT constipation severity			
Moderate	−0.88	0.08	−1.84 to 0.09
Severe to very severe	−1.40	0.15	−3.31 to 0.51
PROMIS anxiety			
UCLA GIT reflux severity			
Moderate	0.53	0.005	0.16 to 0.91
Severe to very severe	1.57	<0.001	1.03 to 2.11
UCLA GIT distention severity			
Moderate	0.82	<0.001	0.38 to 1.26
Severe to very severe	1.24	<0.001	0.75 to 1.72
UCLA GIT diarrhea severity			
Moderate	0.04	0.80	−0.28 to 0.36
Severe to very severe	1.08	0.04	0.07 to 2.11
UCLA GIT constipation severity			
Moderate	0.08	0.63	−0.24 to 0.40
Severe to very severe	0.47	0.18	−0.21 to 1.15
PROMIS depression			
UCLA GIT reflux severity			
Moderate	0.56	0.005	0.17 to 0.95
Severe to very severe	1.70	<0.001	1.12 to 2.27
UCLA GIT distention severity			
Moderate	0.41	0.05	0.01 to 0.82
Severe to very severe	1.54	<0.001	1.04 to 2.04
UCLA GIT diarrhea severity			
Moderate	0.15	0.34	−0.16 to 0.46
Severe to very severe	0.33	0.31	−0.30 to 0.96
UCLA GIT constipation severity			
Moderate	−0.01	0.96	−0.36 to 0.34
Severe to very severe	0.59	0.07	−0.05 to 1.23

* Multivariable population‐averaged panel models using generalized estimating equations with variables including sex, disease duration, SSc subtype, PAH, ILD, digital ulcers, Raynaud phenomenon, myocardial disease, and myositis, with “none to mild” scores used as the reference category. All variables are time varying. PAH was defined as mean pulmonary artery pressure >20 mm Hg, a pulmonary capillary wedge pressure ≤15 mm Hg, and pulmonary vascular resistance >2 Woods units on right‐sided heart catheterization. ILD was defined as the presence of characteristic pulmonary fibrosis on high‐resolution computed tomography of the chest. CI, confidence interval; FACIT‐Fatigue, Functional Assessment of Chronic Illness Therapy–Fatigue; ILD, interstitial lung disease; MCS, mental component score; PAH, pulmonary arterial hypertension; PCS, physical component score; PROMIS, Patient‐Reported Outcomes Measurement Information System; QoL, quality of life; SF‐36, Short Form 36; SHAQ, Scleroderma Health Assessment Questionnaire; SSc, systemic sclerosis; UCLA GIT, University of California, Los Angeles, Scleroderma Clinical Trials Consortium Gastrointestinal Tract 2.0 Questionnaire.

### Physical function and fatigue

Moderate and severe to very severe total UCLA GIT scores were associated with worse physical function (*P* < 0.001; Figure 2) as measured by SHAQ. On multivariable analysis, moderate and severe to very severe scores in all symptom domains were associated with worse physical function (see Table 2), with severe reflux having the most substantial effect (coefficient 7.31, *P* < 0.0001). Moderate and severe to very severe total UCLA GIT scores were associated with worse fatigue (*P* < 0.001; Figure 2) as measured by the FACIT‐Fatigue score. When looking at the effect of individual symptom domains, reflux, and distension once again had the most significant impact on fatigue scores (coefficients −6.33 and −6.00, respectively, *P* < 0.001) with moderate and severe diarrhea also associated with worse fatigue (coefficient −2.07, *P* < 0.05) on multivariable analysis (Table 2).

### Mental well‐being

With respect to QoL domains of the UCLA GIT, social functioning was moderate to severely affected by GIT symptoms in 40% of patients, whereas 48% reported GIT symptoms at least moderately impacted their emotional well‐being (see Supplementary Table 3). On multivariable analysis, moderate and severe to very severe scores in reflux and distension domains were significantly associated with higher PROMIS depression and anxiety scores (*P* < 0.005; see Table 2) with severe diarrhea also associated (*P* < 0.05) with worse anxiety.

### Employment

In our cohort, 342 patients were employed, 112 reported unemployment (97 of whom reported disability preventing work), 287 were retired, and 76 reported home duties (see Supplementary Table 4). Patients with severe total UCLA GIT scores were less likely to be employed compared to those with mild scores (24.9% vs 46.5%; *P* < 0.001). In multivariable GEE analysis, severe to very severe total UCLA GIT scores and severe diarrhea correlated with unemployment (odds ratios 1.66 and 1.33, respectively, *P* < 0.05; Table 3).

**Table 3:** Multivariable analysis of employment status according to UCLA GIT symptoms domain scores*

	Unemployment		
Variable and score	Odds ratio	*P*	95% CI
UCLA GIT total severity			
None to mild	1.00		
Moderate	1.09	0.43	0.88–1.36
Severe to very severe	1.66	0.005	1.17–2.36
UCLA GIT reflux severity			
None to mild	1.00		
Moderate	1.11	0.31	0.91–1.37
Severe to very severe	1.26	0.12	0.94–1.70
UCLA GIT distension severity			
None to mild	1.00		
Moderate	0.95	0.54	0.79–1.13
Severe to very severe	0.98	0.88	0.79–1.22
UCLA GIT diarrhea severity			
None to mild	1.00		
Moderate	1.12	0.14	0.96–1.31
Severe to very severe	1.33	0.04	1.01–1.73
UCLA GIT constipation severity			
None to mild	1.00		
Moderate	1.06	0.42	0.92–1.22
Severe to very severe	0.99	0.95	0.76–1.29

* Multivariable population‐averaged panel models using generalized estimating equations with variables including sex, disease duration, SSc subtype, PAH, ILD, digital ulcers, Raynaud phenomenon, myocardial disease, and myositis with “none to mild” scores used as the reference category. All variables are time varying. PAH was defined as mean pulmonary artery pressure >20 mm Hg, a pulmonary capillary wedge pressure ≤15 mm Hg, and pulmonary vascular resistance >2 Woods units on right‐sided heart catheterization. ILD was defined as the presence of characteristic pulmonary fibrosis on high‐resolution computed tomography of the chest. CI, confidence interval; ILD, interstitial lung disease; PAH, pulmonary arterial hypertension; SSc, systemic sclerosis; UCLA GIT, University of California, Los Angeles, Scleroderma Clinical Trials Consortium Gastrointestinal Tract 2.0 Questionnaire.

### Survival

K‐M curves showed that UCLA GIT scores were not significantly associated with death from all causes or SSc‐related death in our cohort (Supplementary Figures 1 and 2). Patients who died were more likely to be male; be older; and have the diffuse disease subtype, ILD, and PAH (Supplementary Table 5). Total UCLA GIT scores were not significantly associated with death using multivariable Cox proportional hazard regression models (see Supplementary Table 6).

## DISCUSSION

GIT symptoms were common in our cohort, affecting 87% of patients with SSc, and had a significant impact on QoL, physical function, mood, fatigue, and employment. This is the first comprehensive report from the long‐running ASCS and confirms findings from other groups regarding the prevalence and impact of GIT symptoms in SSc.^1^, ^8^, ^20^, ^21^ In a study of 1,902 patients with SSc from 60 countries, GIT symptoms were ranked the second most impactful disease manifestation regarding both QoL and illness severity.^8^ In this study, GIT symptoms were ranked higher than cardiac and pulmonary complications. In another study of 492 patients with SSc, GIT manifestations were found to be an independent determinant of worse health‐related QoL at baseline and a risk factor for worsening QoL over time.^21^

Reflux and distension were the GIT symptoms found to most significantly correlate with poor QoL, physical function, and fatigue (*P* < 0.001) in our cohort. This association remained significant after controlling for demographic and disease features. Our findings are supported by another study of 200 patients with SSc showing severe esophageal dysmotility was associated with lower QoL.^22^ The association between GIT symptoms and worse measures of physical function and fatigue is also in keeping with previous studies.^23^, ^24^, ^25^, ^26^, ^27^, ^28^ Strickland et al reported the absence of upper GIT symptoms was associated with less fatigue and better physical function in their SSc cohort (as measured by FACIT‐Fatigue and the Health Assessment Questionnaire disability index, respectively).^24^ In a study of 944 patients with SSc, Jaeger et al also found a significant association between presence of GIT symptoms (gastric, esophageal, and intestinal) and worse physical function.^23^ The association between upper GIT symptoms and worse fatigue may be due to the effect of GIT symptoms, particularly reflux, on sleep.^12^, ^29^, ^30^, ^31^ A study of 287 patients with SSc showed symptoms of reflux (as measured by the UCLA GIT) were associated with worse sleep quality (as measured by Pittsburgh Sleep Quality Index), an association that remained after controlling for age, sex, and body mass index.^12^

Patients with SSc have a high prevalence of depression.^32^ Some studies have shown an association between GIT symptoms, particularly upper GIT symptoms, and depression in patients with SSc.^7^, ^26^, ^33^ In one study of 152 patients with SSc, worse constipation and reflux symptoms (measured using UCLA GIT 2.0) were associated with worse depression scores as measured by the Center for Epidemiologic Studies Depression Scale (CES‐D) on multivariable analysis, with an increase of 1 U in constipation and reflux scale scores resulting in a 36.5% and 25.9% increase, respectively, in the CES‐D score.^7^ In our cohort, symptoms of reflux and distension were associated with not only higher PROMIS depression scores but also higher anxiety scores. Because symptoms of depression and anxiety have been found to be strongly correlated in SSc,^6^, ^34^ it is unsurprising that GIT symptoms associated with depression were also associated with anxiety in our cohort. Studies in the general population have not only shown an association between mental health and GIT symptoms but also demonstrated the impact of psychological treatment on symptoms.^35^, ^36^, ^37^ Although such studies have yet to be undertaken in SSc, further research investigating the effect of treatment of anxiety and depression on GIT symptoms in SSc may be worthwhile.

Interestingly in our cohort, severe UCLA GIT total scores and diarrhea scores were found to be significantly associated with unemployment (*P* < 0.05). The correlation between total UCLA GIT scores and unemployment is in keeping with our findings of the impact of GIT symptoms on physical function and fatigue. Two previous studies reported that GIT symptoms were associated with unemployment in SSc, although the association did not reach significance on multivariable analysis.^10^, ^38^ To our knowledge, this is the first study to show an association between diarrhea scores and unemployment in SSc, an association that remained after controlling for other demographic and disease features.

Our analysis did not show any relationship between GIT symptoms and death. The majority of our patient population consists of patients with limited disease and long disease duration; therefore, our findings may be impacted by survival bias. Alternatively, this may be because the UCLA GIT has only been collected as part of our database for five years. Therefore, we may not have sufficient longitudinal data to be able to make accurate assumptions in a prevalent cohort. Although GIT involvement has been associated with increased death, this is typically due to complications such as pseudo‐obstruction and malabsorption, which occurred with low frequency in our cohort (<5%).

The majority of participants’ UCLA GIT scores did not change over time, which may be due to the prevalent patient population used in this analysis. The majority of our patients were not incident patients, with an average disease duration of around 14 years. Accordingly, in this cohort, GIT symptoms may represent pre‐existing damage rather than disease activity. Additionally, the UCLA GIT has only been administered in our cohort for the last five years, and the follow‐up may not have been sufficient to see a change in UCLA GIT scores in a cohort of patients with predominantly long‐standing SSc. Alternatively the lack of change in UCLA GIT severity scores may reflect the lack of highly effective treatments for GIT symptoms in SSc.^39^

Moderate FI was only reported in 79 of our patients (9.4%), lower than the previously reported incidence^40^, ^41^, ^42^ of 27% to 38%. The low number of participants with FI may, in part, be due to how this symptom is measured using the UCLA GIT. This questionnaire only includes one question about FI, whereas other studies may use more detailed questionnaires, such as the Jorge‐Wexner FI score.^43^ Alternatively, patient embarrassment in reporting such symptoms may have also contributed to the low numbers of those with FI. Therefore, the reported prevalence of FI in our cohort may be an underestimation.

Our study has several strengths, including the large numbers of patients with SSc from multiple sites around Australia and the use of a validated measure of GIT symptoms, namely the UCLA GIT. Our study is distinguished by the long follow‐up of patients and the availability of a comprehensive suite of other PRO data from validated questionnaires with which we have correlated our UCLA GIT findings. Regarding limitations, having collected the UCLA GIT from patients for only five years may have limited our death analyses because there may not be sufficient longitudinal data to be able to make accurate assumptions in a prevalent cohort. Reassuringly, at most, only 6% of questionnaires included in our analyses had missing data. We did not analyze in detail the demographic and disease features associated with UCLA GIT severity scores or change in UCLA GIT scores over time. Our loss to follow‐up was 17%, which is higher than usual for our cohort and likely due to the period of data collection, which occurred during the COVID‐19 pandemic. However, there was no significant difference between the lost to follow‐up population and those included in this study (Supplementary Table 2).

GIT manifestations are common in patients with SSc and negatively impact QoL, physical function, mood, and employment, but are not directly associated with increased death. Our study highlights the substantial burden associated with GIT symptoms in terms of QoL, physical function, mental health, and employment in SSc. It makes a compelling case for investing more resources in understanding the pathophysiology of GIT involvement and finding novel efficacious treatments.

## AUTHOR CONTRIBUTIONS

All authors contributed to at least one of the following manuscript preparation roles: conceptualization AND/OR methodology, software, investigation, formal analysis, data curation, visualization, and validation AND drafting or reviewing/editing the final draft. As corresponding author, Dr Nikpour confirms that all authors have provided the final approval of the version to be published, and takes responsibility for the affirmations regarding article submission (eg, not under consideration by another journal), the integrity of the data presented, and the statements regarding compliance with institutional review board/Declaration of Helsinki requirements.

## Supporting information


Disclosure form:

**Appendix S1.** Supporting Information.

## References

[1] Gu YS , Kong J , Cheema GS , et al. The immunobiology of systemic sclerosis. Semin Arthritis Rheum 2008;38(2):132–160. 18221988 10.1016/j.semarthrit.2007.10.010

[2] Morrisroe K , Hudson M , Baron M , et al; International Systemic Sclerosis Inception Cohort (INSYNC) collaboration . Determinants of health‐related quality of life in a multinational systemic sclerosis inception cohort. Clin Exp Rheumatol 2018;36(4 suppl 113):53–60. 30183603

[3] Sjogren RW . Gastrointestinal features of scleroderma. Curr Opin Rheumatol 1996;8(6):569–575. 9018461 10.1097/00002281-199611000-00012

[4] Sjogren RW . Gastrointestinal motility disorders in scleroderma. [Review]. Arthritis Rheum 1994;37(9):1265–1282. 7945489 10.1002/art.1780370902

[5] Ebert EC . Gastric and enteric involvement in progressive systemic sclerosis. J Clin Gastroenterol 2008;42(1):5–12. 18097282 10.1097/MCG.0b013e318042d625

[6] Faezi ST , Paragomi P , Shahali A , et al. Prevalence and severity of depression and anxiety in patients with systemic sclerosis: an epidemiologic survey and investigation of clinical correlates. J Clin Rheumatol 2017;23(2):80–86. 28099215 10.1097/RHU.0000000000000428

[7] Bodukam V , Hays RD , Maranian P , et al. Association of gastrointestinal involvement and depressive symptoms in patients with systemic sclerosis. Rheumatology (Oxford) 2011;50(2):330–334. 20884655 10.1093/rheumatology/keq296 PMC3021949

[8] Frantz C , Avouac J , Distler O , et al. Impaired quality of life in systemic sclerosis and patient perception of the disease: a large international survey. Semin Arthritis Rheum 2016;46(1):115–123. 27132536 10.1016/j.semarthrit.2016.02.005

[9] Franck‐Larsson K , Graf W , Rönnblom A . Lower gastrointestinal symptoms and quality of life in patients with systemic sclerosis: a population‐based study. Eur J Gastroenterol Hepatol 2009;21(2):176–182. 19212206 10.1097/MEG.0b013e32831dac75

[10] Sharif R , Mayes MD , Nicassio PM , et al. Determinants of work disability in patients with systemic sclerosis: a longitudinal study of the GENISOS cohort. Semin Arthritis Rheum 2011;41(1):38–47. 21429562 10.1016/j.semarthrit.2011.01.002 PMC3153604

[11] Basta F , Afeltra A , Margiotta DPE . Fatigue in systemic sclerosis: a systematic review. Clinical Exp Rheumatol 2018;36(4 suppl 113):150–160. 29303706

[12] Horsley‐Silva JL , Umar SB , Vela MF , et al. The impact of gastroesophageal reflux disease symptoms in scleroderma: effects on sleep quality. Dis Esophagus 2019;32(5):doy136. 30715227 10.1093/dote/doy136

[13] Steen VD , Medsger TA Jr. Severe organ involvement in systemic sclerosis with diffuse scleroderma. Arthritis Rheum 2000;43(11):2437–2444. 11083266 10.1002/1529-0131(200011)43:11<2437::AID-ANR10>3.0.CO;2-U

[14] Hoffmann‐Vold AM , Volkmann ER . Gastrointestinal involvement in systemic sclerosis: Effects on morbidity and mortality and new therapeutic approaches. J Scleroderma Relat Disord 2019;6(1):37–43. 35382247 10.1177/2397198319891282 PMC8922632

[15] McMahan ZH , Frech T , Berrocal V , et al. Longitudinal assessment of patient‐reported outcome measures in systemic sclerosis patients with gastroesophageal reflux disease ‐ Scleroderma Clinical Trials Consortium. J Rheumatol 2019;46(1):78–84. 30442827 10.3899/jrheum.180004

[16] Khanna D , Hays RD , Park GS , et al. Development of a preliminary scleroderma gastrointestinal tract 1.0 quality of life instrument. Arthritis Rheum. 2007;57(7):1280–1286. 17907224 10.1002/art.22987

[17] van den Hoogen F , Khanna D , Fransen J , et al. 2013 classification criteria for systemic sclerosis: an American College of Rheumatology/European League Against Rheumatism collaborative initiative. Arthritis Rheum 2013;65(11):2737–2747. 24122180 10.1002/art.38098 PMC3930146

[18] Humbert M, Kovacs G, Hoeper MM, et al. 2022 ESC/ERS Guidelines for the diagnosis and treatment of pulmonary hypertension. Eur Respir J. 2023;61(1):2200879. 10.1183/13993003.00879-2022 36028254

[19] Khanna D , Hays R , Maranian P , et al. Reliability, validity, and minimally important differences of the UCLA Scleroderma Clinical Trial Consortium gastrointestinal tract (UCLA SCTC 2.0) instrument. Arthritis Rheum 2009;60(suppl 10):486–487.

[20] Yang H , Xu D , Li MT , et al. Gastrointestinal manifestations on impaired quality of life in systemic sclerosis. J Dig Dis 2019;20(5):256–261. 30838807 10.1111/1751-2980.12720

[21] van Leeuwen NM , Ciaffi J , Liem SIE , et al. Health‐related quality of life in patients with systemic sclerosis: evolution over time and main determinants. Rheumatology (Oxford) 2021;60(8):3646–3655. 33401302 10.1093/rheumatology/keaa827 PMC8328503

[22] Crowell MD , Umar SB , Griffing WL , et al. Esophageal motor abnormalities in patients with scleroderma: heterogeneity, risk factors, and effects on quality of life. Clin Gastr Hepatol 2017;15(2):207–213. 10.1016/j.cgh.2016.08.034 27613260

[23] Jaeger VK , Distler O , Maurer B , et al. Functional disability and its predictors in systemic sclerosis: a study from the DeSScipher project within the EUSTAR group. Rheumatology (Oxford) 2018;57(3):441–450. 28499034 10.1093/rheumatology/kex182

[24] Strickland G , Pauling J , Cavill C , et al. Predictors of health‐related quality of life and fatigue in systemic sclerosis: evaluation of the EuroQol‐5D and FACIT‐F assessment tools. Clin Rheumatol 2012;31(8):1215–1222. 22588647 10.1007/s10067-012-1997-1

[25] Johnson SR , Glaman DD , Schentag CT , et al. Quality of life and functional status in systemic sclerosis compared to other rheumatic diseases. J Rheumatol 2006;33(6):1117–1122. 16622903

[26] Thombs BD , Hudson M , Taillefer SS , et al; Canadian Scleroderma Research Group . Prevalence and clinical correlates of symptoms of depression in patients with systemic sclerosis. Arthritis Rheum 2008;59(4):504–509. 18383422 10.1002/art.23524

[27] Thombs BD , Hudson M , Bassel M , et al; Canadian Scleroderma Research Group . Sociodemographic, disease, and symptom correlates of fatigue in systemic sclerosis: evidence from a sample of 659 Canadian Scleroderma Research Group Registry patients. Arthritis Rheum 2009;61(7):966–973. 19565539 10.1002/art.24614

[28] Assassi S , Leyva AL , Mayes MD , et al; GENISOS Study Group . Predictors of fatigue severity in early systemic sclerosis: a prospective longitudinal study of the GENISOS cohort. PLoS One 2011;6(10):e26061. 22022507 10.1371/journal.pone.0026061 PMC3193535

[29] Sariyildiz MA , Batmaz I , Budulgan M , et al. Sleep quality in patients with systemic sclerosis: relationship between the clinical variables, depressive symptoms, functional status, and the quality of life. Rheumatol Int 2013;33(8):1973–1979. 23370858 10.1007/s00296-013-2680-9

[30] Frech T , Hays RD , Maranian P , et al. Prevalence and correlates of sleep disturbance in systemic sclerosis–results from the UCLA scleroderma quality of life study. Rheumatology (Oxford) 2011;50(7):1280–1287. 21324979 10.1093/rheumatology/ker020 PMC3116211

[31] Figueiredo FP , Aires GD , Nisihara R , et al. Sleep disturbance in scleroderma. J Clin Rheumatol 2021;27(suppl 6):S242–S245. 32568947 10.1097/RHU.0000000000001437

[32] Bragazzi NL , Watad A , Gizunterman A , et al. The burden of depression in systemic sclerosis patients: a nationwide population‐based study. J Affect Disord 2019;243:427–431. 30268959 10.1016/j.jad.2018.09.075

[33] Nietert PJ , Mitchell HC , Bolster MB , et al. Correlates of depression, including overall and gastrointestinal functional status, among patients with systemic sclerosis. J Rheumatol 2005;32(1):51–57. 15630725

[34] Legendre C , Allanore Y , Ferrand I , et al. Evaluation of depression and anxiety in patients with systemic sclerosis. Joint Bone Spine 2005;72(5):408–411. 16214073 10.1016/j.jbspin.2003.11.008

[35] Haug TT , Mykletun A , Dahl AA . Are anxiety and depression related to gastrointestinal symptoms in the general population? Scand J Gastroenterol 2002;37(3):294–298. 11916191 10.1080/003655202317284192

[36] Black CJ , Thakur ER , Houghton LA , et al. Efficacy of psychological therapies for irritable bowel syndrome: systematic review and network meta‐analysis. Gut 2020;69(8):1441–1451. 32276950 10.1136/gutjnl-2020-321191

[37] Ford AC , Lacy BE , Harris LA , et al. Effect of antidepressants and psychological therapies in irritable bowel syndrome: an updated systematic review and meta‐analysis. Am J Gastroenterol 2019;114(1):21–39. 30177784 10.1038/s41395-018-0222-5

[38] Morrisroe K , Huq M , Stevens W , et al; Australian Scleroderma Interest Group (ASIG) . Determinants of unemployment among Australian systemic sclerosis patients: results from a multicentre cohort study. Clin Exp Rheumatol 2016;34(5 suppl 100):79–84. 27463997

[39] Richard N , Gyger G , Hoa S , et al; Canadian Scleroderma Research Group (CSRG), Australian Scleroderma Interest Group (ASIG) . Immunosuppression does not prevent severe gastrointestinal tract involvement in systemic sclerosis. Clin Exp Rheumatol 2021;39(4 suppl 131):142–148. 10.55563/clinexprheumatol/7683pg 34128797

[40] Suresh N , Karanth R , Jayne DG , et al. Fecal incontinence and scleroderma: Pathogenesis and unmet needs. Best Pract Res Clin Rheumatol 2021;35(3):101686. 33895093 10.1016/j.berh.2021.101686

[41] Richard N , Hudson M , Gyger G , et al; on the behalf of Canadian Scleroderma Research Group . Clinical correlates of faecal incontinence in systemic sclerosis: identifying therapeutic avenues. Rheumatology (Oxford) 2017;56(4):581–588. 28013205 10.1093/rheumatology/kew441

[42] Garros A , Marjoux S , Khouatra C , et al. Prevalence of fecal incontinence in a cohort of systemic sclerosis patients within a regional referral network. United European Gastroenterol J 2017;5(7):1046–1050. 10.1177/2050640616688129 PMC5676541 29163972

[43] Jorge JM , Wexner SD . Etiology and management of fecal incontinence. Dis Colon Rectum 1993;36(1):77–97. 8416784 10.1007/BF02050307

