# Supplementary material for: Prevalence and Outcomes of Gastrointestinal Manifestations in an Australian Scleroderma Cohort
Source: Arthritis Care Res (Hoboken). 2024 Oct 4;76(12):1686–95. doi: 10.1002/acr.25426 (PMC11605790; doi:10.1002/acr.25426)
Supplement: Supplementary file 2 — Appendix S1. Supporting Information. [file ACR-76-1686-s002.docx]

Supplementary Table of Contents

Supplementary table 1: Number of UCLA GIT surveys completed page 2

Supplementary table 2: Demographic and clinical characteristics by lost to follow up status page 2

at last review

Supplementary table 3: Participant UCLA GIT score by symptom domain page 3

Supplementary table 4: Employment status according to UCLA GIT total score page 3

Supplementary table 5: Demographic and clinical characteristics by mortality status at last page 4

review

Supplementary table 6: Multivariable cox proportional hazard regression model for survival page 4

according to UCLA GIT total score

Supplementary figure 1: Kaplan-Meier survival estimates of all-cause mortality by UCLA

GIT total score page 4

Supplementary figure 2: Kaplan-Meier survival estimates of scleroderma associated mortality

by UCLA GIT total score page 5

### Supplementary table 1 - Number of UCLA GIT surveys completed

| Number of surveys completed | n (%) |
| --- | --- |
| 1 survey | 244 (26.9%) |
| 2 surveys | 212 (23.4%) |
| 3 surveys | 168 (18.5%) |
| 4 surveys | 198 (21.8%) |
| 5 surveys | 85 (9.4%) |

UCLA – University of California and Los Angeles, GIT- Gastrointestinal, n- number of participants

**Supplementary Table 2**- Demographic and clinical characteristics by lost to follow up status at last review

| Characteristics  (mean ± SD or n(%)) | Current  (n=656) | | Lost to follow up  (n=144) | p |
| --- | --- | --- | --- | --- |
| Female | | 571 (87.4%) | 125 (86.8%) | 0.84 |
| Age at review | | 61.3 (12.6) | 60.0 (14.5) | 0.28 |
| Disease duration, years | | 13.5 (7.2-21.7) | 13.1 (5.7-22.5) | 0.53 |
| Disease subtype  Limited  Diffuse | | 479 (78.0%) 135 (22.0%) | 101 (77.7%)  29 (22.3%) | 0.94 |
| Digital Ulcers* | | 183 (27.9%) | 32 (22.2%) | 0.16 |
| Calcinosis* | | 71 (11.4%) | 12 (8.9%) | 0.40 |
| Myositis* | | 61 (9.6%) | 9 (7.0%) | 0.35 |
| Myocardial disease* | | 47 (7.4%) | 7 (5.3%) | 0.38 |
| PAH | | 31 (4.7%) | 6 (4.2%) | 0.77 |
| ILD | | 192 (29.3%) | 42 (29.2%) | 0.98 |
| Pseudo obstruction | | 15 (2.3%) | 3 (2.2%) | 0.93 |
| Malabsorption | | 21 (3.4%) | 2 (1.9%) | 0.40 |
| UCLA GIT total score  Mild  Moderate  Severe | | 404 (61.6%)  155 (23.6%)  97 (14.8%) | 86 (59.7%)  40 (27.8%)  18 (12.5%) | 0.51 |

Abbreviations: UCLA- University of California and Los Angeles, GIT – gastrointestinal, SD- standard deviation, n- number, PAH- pulmonary arterial hypertension, ILD – interstitial lung disease

*Ever recorded during follow up

PAH defined as mean pulmonary artery pressure (mPAP)>20mmHg and a pulmonary capillary wedge pressure (PCWP) <15mmHg and pulmonary vascular resistance (PVR) >2 Woods units on right heart catheter

ILD defined as the presence of characteristic pulmonary fibrosis on HRCT of the chest

**Supplementary table 3**– Participant UCLA score by symptom domain

| UCLA GIT scores |  | n (%) |
| --- | --- | --- |
| Reflux | None-to-Mild | 389 (45.0%) |
|  | Moderate | 254 (29.4%) |
|  | Severe-to Very Severe | 221 (25.6%) |
| Distention/Bloating | None-to-Mild | 463 (53.7%) |
|  | Moderate | 121 (14.0%) |
|  | Severe-to Very Severe | 278 (32.3%) |
| Diarrhoea | None-to-Mild | 352 (40.9%) |
|  | Moderate | 349 (40.5%) |
|  | Severe-to Very Severe | 160 (18.6%) |
| Constipation | None-to-Mild | 410 (48.0%) |
|  | Moderate | 319 (37.3%) |
|  | Severe-to Very Severe | 126 (14.7%) |
| Soilage/ Faecal incontinence | None-to-Mild | 764 (90.6%) |
|  | Moderate | 79 (9.4%) |
|  | Severe-to Very Severe | 0 (0.0%) |
| Emotional Well-being | None-to-Mild | 574 (66.8%) |
|  | Moderate | 121 (14.1%) |
|  | Severe-to Very Severe | 164 (19.1%) |
| Social functioning | None-to-Mild | 523 (60.6%) |
|  | Moderate | 226 (26.2%) |
|  | Severe-to Very Severe | 114 (13.2%) |
| Total GIT score | None-to-Mild | 445 (52.2%) |
|  | Moderate | 231 (27.1%) |
|  | Severe-to Very Severe | 177 (20.8%) |

UCLA – University of California and Los Angeles, GIT- Gastrointestinal, n- number of participants

**Supplementary Table 4**-Employment status according to UCLA GIT total score

|  | |  | Severe-to-Very Severe (n=177) | Moderate (n=231) | None-to-Mild (n=445) |
| --- | --- | --- | --- | --- | --- |
| Employed | | |  |  |  |
|  | Full time | | 17 (9.6%) | 45 (19.4%) | 120 (27.0%) |
|  | Part time | | 26 (14.7%) | 46 (19.9%) | 83 (18.6%) |
|  | Student | | 1 (0.6%) | 0 (0.0%) | 4 (0.9%) |
| Unemployed | | |  |  |  |
|  | Disability preventing work | | 38 (22.2%) | 30 (13.8%) | 29 (6.8%) |
|  | Unemployed | | 4 (2.3%) | 1 (0.5%) | 10 (2.3%) |
| Other | | |  |  |  |
|  | Retired | | 63 (36.8%) | 73 (33.6%) | 151 (35.2%) |
|  | Home duties | | 22 (12.9%) | 22 (10.1%) | 32 (7.5%) |

UCLA – University of California and Los Angeles, GIT- Gastrointestinal

**Supplementary Table 5** - Demographic and clinical characteristics by mortality status at last review

| Characteristics  (mean ± SD or n(%)) | Current  (n=656) | | Deceased  (n=53) | p |
| --- | --- | --- | --- | --- |
| Female | | 571 (87.4%) | 37 (69.8%) | <0.0001 |
| Age at review (years) | | 61.3 (12.6) | 67.4 (10.2) | 0.001 |
| Disease duration, years | | 13.5 (7.2-21.7) | 15.2 (7.7-24.6) | 0.41 |
| Disease subtype  Limited  Diffuse | | 479 (78.0%)  135 (22.0%) | 31 (60.8%)  20 (39.2%) | 0.005 |
| Digital Ulcers* | | 183 (27.9%) | 24 (45.3%) | 0.008 |
| Calcinosis* | | 71 (11.4%) | 5 (9.6%) | 0.70 |
| Myositis* | | 61 (9.6%) | 6 (11.3%) | 0.69 |
| Myocardial disease* | | 47 (7.4%) | 7 (13.5%) | 0.12 |
| PAH | | 31 (4.7%) | 21 (39.6%) | <0.001 |
| ILD | | 192 (29.3%) | 30 (56.6%) | <0.001 |
| Pseudo obstruction | | 15 (2.3%) | 1 (1.9%) | 0.84 |
| Malabsorption | | 21 (3.4%) | 1 (2.0%) | 0.59 |
| UCLA GIT total score  None to Mild  Moderate  Severe to Very Severe | | 404 (61.6%)  155 (23.6%)  97 (14.8%) | 33 (62.3%)  11 (20.8%)  9 (17.0%) | 0.85 |

Abbreviations: UCLA- University of California and Los Angeles, GIT – gastrointestinal, SD- standard deviation, n- number, PAH- pulmonary arterial hypertension, ILD – interstitial lung disease

*Ever recorded during follow up

PAH defined as mean pulmonary artery pressure (mPAP)>20mmHg and a pulmonary capillary wedge pressure (PCWP) <15mmHg and pulmonary vascular resistance (PVR) >2 Woods units on right heart catheter

ILD defined as the presence of characteristic pulmonary fibrosis on HRCT of the chest

**Supplementary Table 6**- Multivariable cox proportional hazard regression model for survival according to UCLA GIT total score

| Variable | Value | Hazard Ratio | p | 95% CI |
| --- | --- | --- | --- | --- |
| UCLA GIT score severity | None-to-Mild | 1.00 |  |  |
|  | Moderate | 0.95 | 0.89 | 0.47 - 1.91 |
|  | Severe-to Very Severe | 0.92 | 0.83 | 0.43 - 1.98 |
| Disease subclassification | Diffuse | 1.00 |  |  |
|  | Limited | 1.00 | 0.99 | 0.47 - 2.15 |
| Gender | Male | 1.00 |  |  |
|  | Female | 0.59 | 0.15 | 0.29 - 1.21 |
| PAH | Yes | 9.44 | <0.001 | 5.04 - 17.67 |
| ILD* | None | 1.00 |  |  |
|  | Mild(<20%) | 1.68 | 0.17 | 0.80 - 3.51 |
|  | Moderate(20-30%) | 2.50 | 0.03 | 1.10 - 5.70 |
|  | Severe(>30%) | 2.46 | 0.09 | 0.87 - 6.93 |
| Myocardial disease | Definite or suspected | 0.92 | 0.83 | 0.41 - 2.04 |
| Myositis** | Definite or suspected | 1.10 | 0.83 | 0.44 - 2.75 |
| Digital ulcers** | Yes | 1.08 | 0.84 | 0.52 – 2.25 |

UCLA – University of California and Los Angeles, GIT- Gastrointestinal, PAH- pulmonary arterial hypertension, ILD – interstitial lung disease

*Worst ever severity recorded during follow up

**Ever recorded during follow up

PAH defined as mean pulmonary artery pressure (mPAP)>20mmHg and a pulmonary capillary wedge pressure (PCWP) <15mmHg and pulmonary vascular resistance (PVR) >2 Woods units on right heart catheter

ILD defined as the presence of characteristic pulmonary fibrosis on HRCT of the chest

**Supplementary Figure 1** – Kaplan-Meier survival estimates of all-cause mortality by UCLA GIT total score


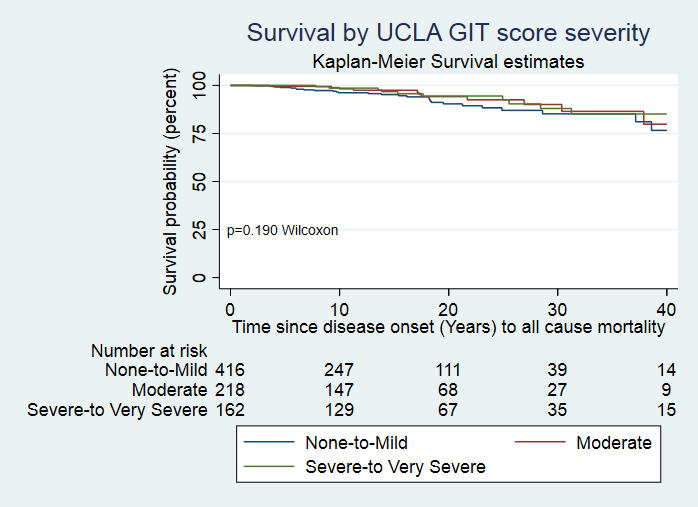


UCLA – University of California and Los Angeles, GIT- Gastrointestinal, n- number of participants

**Supplementary Figure 2** – Kaplan-Meier survival estimates of scleroderma associated mortality by UCLA GIT total score


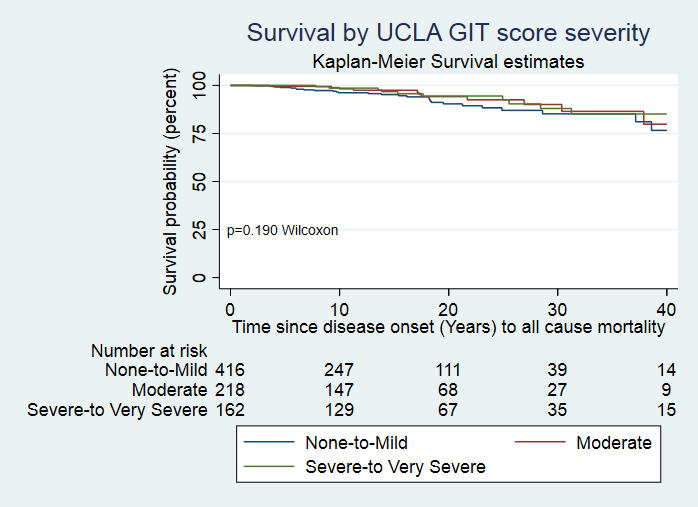


UCLA – University of California and Los Angeles, GIT- Gastrointestinal, n- number of participants
